# Automated and manual microscopic analyses for leukocyte differential counts in exudative pleural effusions: Real-world disagreement and clinical application

**DOI:** 10.1097/MD.0000000000030611

**Published:** 2022-09-16

**Authors:** Jaehee Lee, Yu Kyung Kim, Ji Eun Park, Yong Hoon Lee, Sun Ha Choi, Hyewon Seo, Seung Soo Yoo, Shin Yup Lee, Seung-Ick Cha, Jae Yong Park, Chang Ho Kim

**Affiliations:** a Department of Internal Medicine, School of Medicine, Kyungpook National University, Daegu, South Korea; b Department of Clinical Pathology, School of Medicine, Kyungpook National University, Daegu, South Korea.

**Keywords:** automated hemocytometer analyzer, cytospin, differential leukocyte counts, manual microscopic analysis, pleural effusion

## Abstract

Differential leukocyte counts of pleural fluid are routinely recommended for the early diagnosis and management of exudative pleural effusions. Rapid automated cellular analysis agrees strongly with standard manual microscopic counts and has become a reality in many clinical laboratories. However, discordant results sometimes observed between automated and manual analyses raise concern about using automated analysis to aid prompt differential diagnosis. This study aimed to evaluate the real-world disagreement between automated and manual leukocyte analyses in exudative pleural effusions and to investigate whether the discordant results occur in specific cellular ranges or randomly. We conducted a retrospective study of patients who were diagnosed with parapneumonic pleural effusions (PPE), tuberculous pleural effusions (TPE), and malignant pleural effusions (MPE) between September 2018 and December 2020. Differential and predominant leukocyte counts were performed using an automated XN-350 analyzer with a two-part differential count consisting of polymorphonuclear (PMN) and mononuclear (MN) leukocytes and a manual method with Wright-stained cytospin slides. We compared the two methods on cases of 109 PPEs, 50 TPEs, and 116 MPEs. Although the overall correlation between the two methods for differential leukocyte counts was excellent, there were etiologic variations; MPEs showed a lower correlation compared to PPEs and TPEs. Automated-PMN predominance almost corresponded to manual cytospin-neutrophilic predominance. In contrast, ~10% of the automated-MN predominance did not correspond with the cytospin-lymphocytic predominance. These discrepancies occurred most in the automated-MN% range of 51% to 60%, followed by 61% to 70%. The PMN% range ≥50% and <30% on the automated analysis reliably corresponds to the neutrophilic and lymphocytic predominance, respectively. However, the MN% range of 51% to 70% may not coincide with lymphocytic predominance on manual cytospin analysis. This range leaves the potential cause of exudative pleural effusions open.

## 1. Introduction

Cellular analysis is routinely performed to guide the differential diagnosis of exudative pleural effusions. The accurate and timely analysis of pleural fluid cellular components can facilitate prompt patient management.^[1]^ Manual microscopic counting with chamber counting and cytospin analysis is regarded as the gold standard method.^[2,3]^ However, it has several drawbacks, including imprecision, high inter-observer variability, poor reproducibility, and long turnaround time.^[4,5]^ The limitations of manual cell counts and the adaptability of automated hematology analyzers to body fluids have led to greater interest, innovation, and consequently use of automated pleural fluid analysis in many laboratories.^[2,6]^

Several studies have demonstrated an excellent correlation between automated cellular analysis and the manual method regarding total nucleated cells (TNC) and overall differential leukocyte counts of pleural fluid.^[7–13]^ According to Food and Drug Administration approval, automated methods classify pleural fluid leukocytes into a two-part differential count consisting of polymorphonuclear (PMN) and mononuclear (MN) leukocytes.^[6]^ Regarding pleural fluid cellular predominance for narrowing differential diagnosis, PMN predominance shown by the automated method is usually compatible with neutrophilic predominance seen with the manual method. Likewise, MN predominance in a pleural effusion is regarded as lymphocytic-predominant. The correlation between these white blood cell (WBC) parameters is generally excellent. However, disagreements between automated and cytospin analysis-derived predominant leukocytes are sometimes seen in the “real-world” clinical practice. These discordant results may lead to concern about using automated analysis for prompt differential diagnosis. Thus, it may be helpful to understand the property of discrepancy between the two methods according to the different effusion etiologies. In addition, there are little data about whether these discordant results are observed in specific cellular range or randomly.

This study aimed to compare automated and manual pleural fluid leukocyte parameters, in exudative pleural effusions of three etiologies, commonly encountered in clinical practice. Furthermore, the study investigated whether the discordant results occur in specific cellular ranges.

## 2. Materials and Methods

### 2.1. Effusion etiology and diagnostic criteria

This study was conducted at Kyungpook National University Hospital, a tertiary referral hospital in South Korea, using the electronic Pleural Diseases Database. We retrospectively reviewed the data of all consecutive patients who were diagnosed with parapneumonic pleural effusions (PPE), tuberculous pleural effusions (TPE), and malignant pleural effusions (MPE) between September 2018 and December 2020, when automated cellular analysis of body fluid was performed. PPE was defined as exudative pleural effusion associated with bacterial pneumonia, lung abscesses, or bronchiectasis. TPE was diagnosed if there was either a positive culture or polymerase chain reaction for *Mycobacterium tuberculosis* in pleural fluid, pleural tissue, or respiratory specimens (confirmed); or pathologically, there was pleural granulomatous inflammation with no evidence of other granulomatous disease and good response to anti-tuberculosis chemotherapy (probable). MPE was diagnosed if malignant cells were identified in the pleural fluid or pleural tissue (confirmed); or if an exudative pleural effusion was shown in patients with a confirmed malignancy, after ruling out other benign causes (probable).

### 2.2. Sample processing and results comparison

Fresh samples of pleural fluid referred to the clinical laboratory for cell counts and leukocyte differential analysis from inpatient wards were simultaneously assessed by automated Sysmex XN-350 (Sysmex, Kobe, Japan) and manual cytospin analysis. TNC, leukocytes with PMN and MN differential count, and high-fluorescence body fluid (HF-BF) cells were counted with the XN-350 body-fluid mode within 2 hours. For cytospin analysis, slides were prepared by cytocentrifugation of the samples followed by Wright staining. At least 200 cells were counted at 400× magnification. Leukocytes were classified and represented as neutrophils%, lymphocytes%, monocytes/macrophages%, eosinophils%, and basophils%.^[13]^

The correlation between the automated and manual-cytospin leukocyte parameters was assessed according to the exudative pleural effusion etiologies. In addition, the results of PMN% and MN% on the XN-350 analysis were compared with those of neutrophils% and lymphocytes% on the cytospin analysis. PMN-predominant fluid was defined as effusion with PMN% more than MN% with the XN-350 method, with the reverse being MN-predominant fluid. In addition, neutrophil-predominant effusion was defined as effusion with neutrophil% more than lymphocyte% on the cytospin analysis, with the reverse being a lymphocyte-predominant effusion.

The basic performance characteristics of the XN-350, including precision, carryover, and linearity, were not evaluated because it has been done in many previous studies with excellent results.^[7–13]^ Likewise, TNC counts with the Neubauer chamber were replaced with those of the automated method because there was also an excellent correlation between the two methods. Further, it is of less value in the interpretation of pathologic effusions in contrast to transudate.^[6]^ The methodologies used in this study followed the Clinical Laboratory Standards Institute guideline (CLSI H56-A).^[2]^ The study protocols were approved by the Institutional Review Board (2021-06-034) of Kyungpook National University Hospital. Informed consent was waived because of the retrospective nature of the study.

### 2.3. Statistical analysis

Statistical analyses were performed using IBM SPSS Statistics for Windows version 22.0 (IBM Corp., Armonk, NY). Continuous variables are expressed as median (interquartile range), and differences between groups were analyzed using the *t* test or Kruskal–Wallis test. Categorical variables are expressed as absolute values and percentages and were analyzed using the *χ*^2^ test or Fisher’s exact test. The correlation between automated and cytospin analyses for differential leukocyte parameters were assessed using Pearson’s correlation coefficient. Variables with *P* values < .05 were considered statistically significant.

## 3. Results

A total of 275 patients with PPE (n = 109), TPE (n = 50; of which 46 confirmed, 4 probable), and MPE (n = 116; of which 84 confirmed, 32 probable) were analyzed with the automated and cytospin methods for differential leukocyte counts of pleural fluid. The patient demographics and baseline pleural fluid cell counts measured with the automated method are provided in Table 1. The median age of the patients was 70 years. As expected, the PMN% was significantly higher in the PPEs than in the TPEs and MPEs, whereas the MN% was significantly lower in the PPEs. In addition, HF-BF cells/μL and HF-BF%/100 WBC were significantly higher in the MPEs than in the two benign effusions.

**Table 1:** Demographics and baseline pleural fluid cell counts measured by the automated method in patients with PPE, TPE, and MPE.

Variable	PPE (n = 109)	TPE (n = 50)	MPE (n = 116)	*P* value
Demographic				
Age, yr	70 (61–79)	74 (65–81)	74 (65–81)	.138
Male	86 (79)	32 (64)	74 (64)	.029
Pleural fluid				
Total nucleated cells, /μL	7144 (1990–23637)	2545 (1844–4158)	1655 (923–3022)	<.001
White blood cells, /μL	7133 (1757–23625)	2488 (1812–4028)	1539 (754–2793)	<.001
PMN%	81 (60–90)	6 (2–17)	8 (4–21)	<.001
MN%	19 (10–40)	94 (83–98)	92 (79–96)	<.001
HF-BF cells, /μL	10 (2–97)	26 (10–73)	102 (40–234)	<.001
HF-BF cells %, /100 WBC	0.2 (0–0.7)	1.1 (0.4–3.4)	6.3 (3.1–18.2)	<.001

Data are expressed as the number (%) or median (IQR).

HF-BF = high fluorescence-body fluid, IQR = interquartile range, MN = mononuclear leukocytes, MPE = malignant pleural effusion, PMN = polymorphonuclear leukocytes, PPE = parapneumonic pleural effusion, TPE = tuberculous pleural effusion, WBC = white blood cells.

### 3.1. Correlation between automated and manual cytospin analyses for leukocyte differential counts

Table 2 shows the correlation between the automated and manual cytospin parameters for WBC differential counts in all and individual pleural effusions. The correlation coefficients (*r*) between PMN% determined by the automated method and neutrophils% by the cytospin method in the PPEs, TPEs, and MPEs were 0.891, 0.903, and 0.862, respectively. The values for automated-MN% and cytospin-lymphocytes% in the PPEs, TPEs, and MPEs were 0.825, 0.841, and 0.600, respectively. The correlation between automated-PMN% and cytospin-neutrophils% was better than between automated-MN% and cytospin-lymphocytes% in all three types of pleural effusion. Correlations were improved when using the combined parameters ([neutrophils + eosinophils + basophils] or [lymphocytes + monocytes + macrophages]) of cytospin analysis than single neutrophils or lymphocytes compared with their corresponding automated-PMN% and MN%, especially in MPEs.

**Table 2:** Pearson’s correlation coefficients (*r*) between automated and manual cytospin analyses for leukocyte differential counts in the different etiologies of exudative pleural effusion.

Automated vs cytospin	Total (n = 275)	PPE (n = 109)	TPE (n = 50)	MPE (n = 116)
PMN% vs NE%	0.967	0.891	0.903	0.862
PMN% vs (NE + EO + BA)%	0.972	0.896	0.902	0.910
MN% vs LY%	0.866	0.825	0.841	0.600
MN% vs (LY + MO + MA)%	0.972	0.893	0.905	0.919

BA = basophils, EO = eosinophils, LY = lymphocytes, MA = macrophages, MN = mononuclear leukocytes, MO = monocytes, MPE = malignant pleural effusion, NE = neutrophils, PMN = polymorphonuclear leukocytes, PPE = parapneumonic pleural effusion, TPE = tuberculous pleural effusion.

### 3.2. Disagreement between automated-PMN (or MN) and manual cytospin-neutrophils (or lymphocytes) predominance

The real-world disagreement between automated-PMN and cytospin-neutrophils predominance and between automated-MN and cytospin-lymphocytes predominance, respectively, was analyzed. In 109 PPEs, all 94 (100%) automated-PMN predominance resulted in cytospin-neutrophils predominance, whereas 9 (60%) out of 15 automated-MN predominance resulted in cytospin-lymphocytes predominance (Table 3). In 50 TPEs, all four (100%) automated-PMN predominance showed cytospin-neutrophils predominance, and 45 (98%) out of 46 automated-MN predominance were cytospin-lymphocytes predominance. In 116 MPEs, seven (88%) out of eight automated-PMN predominance were cytospin-neutrophils predominance, and 98 (91%) out of 108 automated-MN predominance were cytospin-lymphocytes predominance. Like the correlation coefficients, the overall agreement between automated and cytospin methods for predominant leukocytes was better in PMN predominance than in MN predominance, and TPEs showed better agreement than PPEs and MPEs.

**Table 3:** Overall agreement of neutrophils and lymphocytes predominance on manual cytospin analysis based on PMN and MN predominance, respectively, measured by the automated method in the different etiologies of exudative pleural effusion.

Etiology	Neutrophils/PMN	Lymphocytes/MN	Total
PPE (n = 109)	94/94 (100)	9/15 (60)	103/109 (94)
TPE (n = 50)	4/4 (100)	45/46 (98)	49/50 (98)
MPE (n = 116)	7/8 (88)	98/108 (91)	105/116 (91)
Total (n = 275)	105/106 (99)	152/169 (90)	257/275 (93)

Data are expressed as the number (%).

MN = mononuclear leukocytes, MPE = malignant pleural effusion, PMN = polymorphonuclear leukocytes, PPE = parapneumonic pleural effusion, TPE = tuberculous pleural effusion.

### 3.3. Discordant results between automated and manual cytospin analyses for predominant leukocytes according to the automated-PMN% range

Most pleural effusions (93%) showed concordant results between automated- and cytospin-predominance for the two-part differential leukocytes (PMN vs neutrophils and MN vs lymphocytes), and a few (7%) cases resulted in discrepancy (Table 3). Therefore, we assessed whether these discordant results occurred in a certain specific PMN% range of automated analysis or randomly.

**Figure 1.**
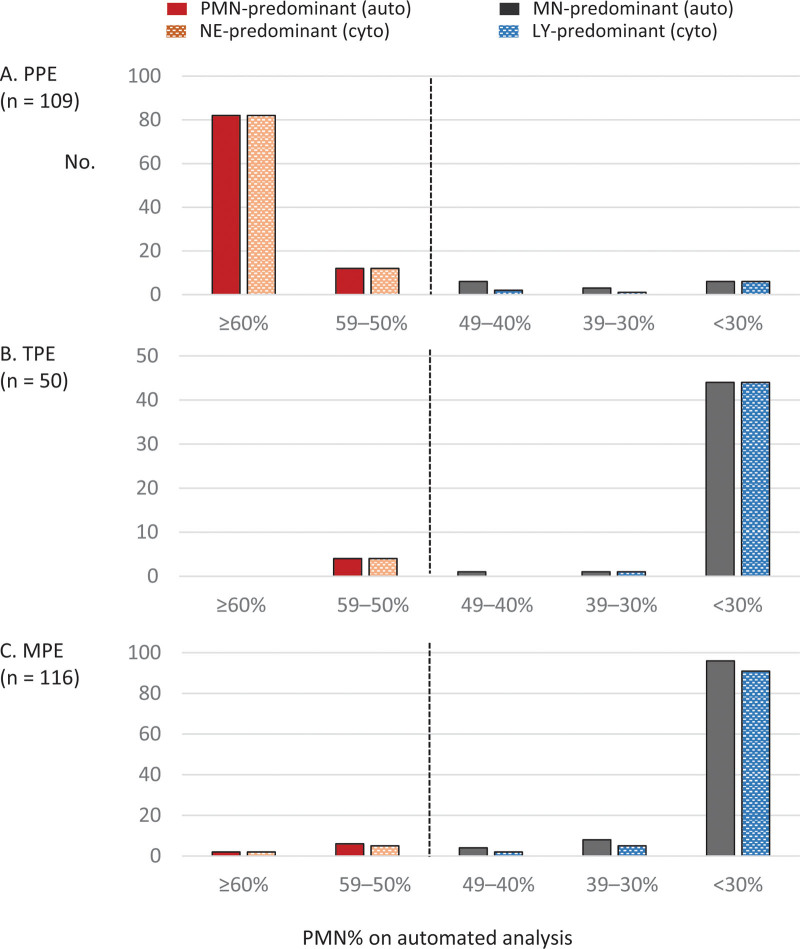
Agreement of predominant leukocytes between automated and manual cytospin analyses according to the PMN% range of automated cellular analysis of PPE (A), TPE (B), and MPE (C). Auto = automated analysis, cyto = cytospin analysis, LY = lymphocytes, MN = mononuclear leukocytes, MPE = malignant pleural effusion, NE = neutrophils, PMN = polymorphonuclear leukocytes, PPE = parapneumonic effusion, TPE = tuberculous pleural effusion.

In PPEs, when the automated-PMN% range was ≥50% or <30% (i.e., MN% ≥70%), all cytospin analyses showed concordant neutrophilic or lymphocytic predominance, respectively (Fig. 1). All six discordant results (Table 3) were observed in the automated-PMN% range of 30% to 49% (i.e., MN% of 51%–70%). Likewise, these characteristics were shown even in TPEs and MPEs. One discordant TPE occurred in the automated-PMN% range of 40% to 49%, and this case with an automated-PMN% of 45% showed cytospin-neutrophils predominance of 61%. In MPEs with more discordant cases, the PMN% range of 40% to 49% (i.e., MN% of 51%–60%) was the most common discordant sector (two out of four cases), followed by the PMN% range of 30% to 39% (three out of eight cases) and < 30% (5 of 96 cases) sectors. One discordant MPE, which was found in the PMN% range of 50% to 59%, showed hydropneumothorax on chest X-ray before diagnostic thoracentesis. Thus, though the automated method showed a PMN predominance of 56%, cytospin analysis revealed neutrophil counts of 1% and a predominant eosinophil count of 66%.

**Figure 2.**
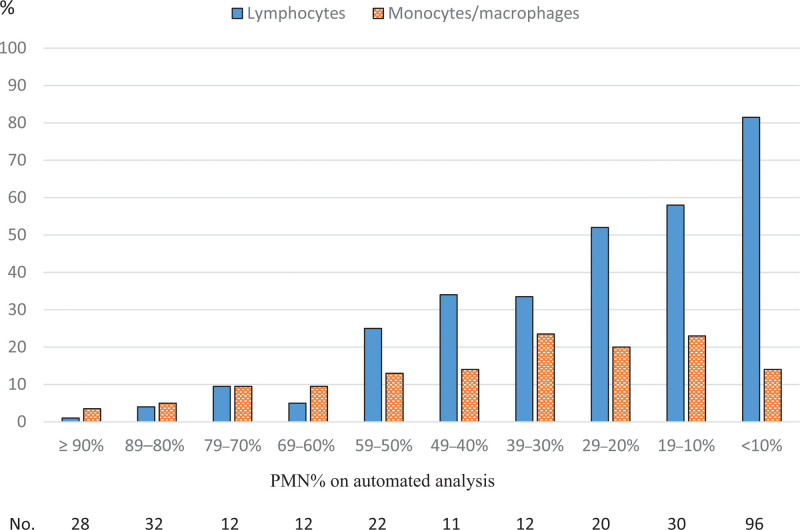
Median percentages of lymphocytes and monocytes/macrophages on manual cytospin analysis according to the polymorphonuclear leukocyte (PMN)% range of automated cellular analysis of exudative pleural fluid including parapneumonic (n = 109), tuberculous (n = 50), and malignant (n = 116) pleural effusions.

Figure 2 shows the percentages of lymphocytes and monocytes (including macrophages) measured by cytospin analysis according to the PMN% range of the automated method in the total population. Lymphocytes were increased according to the decline of automated-PMN%, whereas monocytes/macrophages did not show similar increments. These characteristics were similar in the three etiologies of exudative pleural effusion (data not shown).

## 4. Discussion

The primary findings of this study were as follows: although the overall correlation between automated and manual cytospin methods for differential leukocyte counts in exudative pleural effusions was very strong or strong, there was some variation according to etiology; the correlation between automated-PMN% and manual cytospin-neutrophils% was better than between automated-MN% and cytospin-lymphocytes% for the three different etiologies; the discordant results between automated and manual cytospin predominant leukocytes were primarily observed between automated-MN and manual cytospin-lymphocytes predominance; these discrepancies were most commonly found in the automated-PMN% range of 40% to 49%, followed by 30% to 39%, in all three types of exudative pleural effusion.

Rapid and reliable predominant cell determination of pleural fluid is important in the early presumptive diagnosis and management of patients with exudative pleural effusion. In clinical practice, the automated method is faster than the manual microscopic cytospin method.^[6]^ In addition, previous studies have shown that the overall correlation between automated and manual methods is excellent, except for cerebrospinal fluid.^[7–13]^ Furthermore, HF-BF cells, with the automated method, provide additional helpful information in screening malignancy.^[13–16]^ Thus, real-world automated analysis may be more clinically useful. This study also showed a strong overall correlation between the two methods. However, our study showed that there might be some difference according to the etiology of the pleural effusion. These results may be attributable to the different cellular composition that depends upon the pleural insult by the underlying disease^[17–20]^ in addition to the time of thoracentesis. TPE seems to have a relatively simple, more homogenous lymphocytic effusion in terms of pleural injury, whereas MPE appears to show more complex cellular composition due to the heterogeneous microenvironment of the pleura.

The correlation between automated-MN% and cytospin-lymphocytes% was lower than between automated-PMN% and cytospin-neutrophils% in all three etiologies of exudative pleural effusion. The better correlation between automated-PMN% and cytospin-neutrophils% suggests that the contribution of eosinophils and basophils was less to the PMN%, compared with the contribution of monocyte and macrophage to MN%. In particular, the effect of monocytes/macrophages was prominent in MPE, which is in accordance with previous studies.^[18–20]^ Thus, the correlation between automated-MN% and cytospin-lymphocytes% was the lowest in MPEs.

Nearly all automated-PMN predominance coincided with cytospin-neutrophils predominance. Therefore, it is likely that automated-PMN-predominant results are reliably applicable to neutrophil-predominant effusions, except for cases with pleural fluid eosinophilia, as shown in one MPE case in this study. In contrast, ~10% of automated-MN predominance samples were not consistent with lymphocytes predominance on cytospin analysis.

The discrepancy between the two methods for dichotomous predominant leukocytes was primarily observed in the PMN% range of 30% to 49% (i.e., MN% of 51%–70%). These findings were similar in all three types of exudative pleural effusion (Fig. 1). In the PMN% range <30% (i.e., MN% ≥70%), lymphocytes alone reached >50% among leukocytes (Fig. 2). Thus, like automated-PMN predominance, almost automated-PMN% <30% (i.e., MN% ≥70%) led to lymphocytic predominance. In contrast, the PMN% range of 30% to 49% had a higher proportion of monocytes/macrophages, which accounted for ~50% of the cytospin-MN% (sum of lymphocytes% and monocytes/macrophages%). Thus, it may be possible that the proportion of neutrophils was greater than that of lymphocytes on cytospin analysis in some cases. This cellular distribution might correspond to the subacute rather than acute or chronic stage.^[21–23]^ Thus, these phenomena could be observed in the subacute stage of any exudative pleural effusion. These relationships need to be considered in the clinical application of automated analysis. We did not evaluate the difference of lymphocytes and MN predominance in clinical significance for managing exudative pleural effusions; they may have a similar impact. Further study is warranted to investigate this.

This study had some limitations. First, it was a single-center retrospective study and may be subject to selection bias. However, the cellular analysis of pleural fluid is unlikely to have been affected by retrospective review. Second, the reporting of cell counts on manual cytospin analysis may be different between laboratories. For example, some laboratories include mesothelial cells or macrophages in the differential count as MNs, whereas others exclude them.^[6]^ Our laboratory did not separate monocytes and macrophages in cytospin leukocyte differential counts.

In conclusion, this study evaluated the relationship between automated and manual microscopic (cytospin) analyses for WBC differential counts of pleural fluid. We compared the two methods in one neutrophilic (PPE) and two lymphocytic (TPE and MPE) predominant exudative effusions. Overall, the automated method, with its advantages of consistency and short turnaround time, showed strong agreement with the manual cytospin method. The PMN% range ≥50% and <30% of the automated cellular analysis can be reliably applicable to neutrophilic and lymphocytic predominance, respectively. However, MN predominance on automated analysis, especially in the PMN% range of 49% to 30% (i.e., MN% of 51%–70%), may not coincide with lymphocytic predominance on the manual cytospin analysis, and thus any cause of exudative pleural effusions may be possible.

## Author contributions

**Conceptualization:** Chang Ho Kim.

**Data curation:** Jaehee Lee, Yu Kyung Kim.

**Formal analysis:** Jaehee Lee, Chang Ho Kim.

**Methodology:** Ji Eun Park.

**Supervision:** Jae Young Park.

**Writing – original draft:** Chang Ho Kim.

**Writing – review & editing:** Young Hoon Lee, Sun Ha Choi, Hyewon Seo, Seung Soo Yoo, Shin Yup Lee, Seung-Ick Cha.
